# Pneumothorax following ERCP: Report of Two Cases with Different Pathophysiology

**DOI:** 10.1155/2013/206564

**Published:** 2013-06-24

**Authors:** Kyriakos Neofytou, Athanasios Petrou, Constantinos Savva, Christos Petrides, Charalampos Andreou, Evangelos Felekouras, Sakis Loizou

**Affiliations:** ^1^Department of Surgery, Nicosia Government Hospital, Palaios Dromos Lefkosias-Lemesou, No. 215, Strovolos, 2029 Nicosia, Cyprus; ^2^Department of Surgery, Aretaeio Private Hospital, Andrea Avraamides Street, Dasoupoli, 2014 Nicosia, Cyprus; ^3^First Department of Surgery, Laiko General Hospital, University of Athens Medical School, 115 27 Athens, Greece

## Abstract

In the last thirty years, the widespread use of endoscopic retrograde cholangiopancreatography (ERCP) has radically changed the management of patients with diseases of the extrahepatic biliary tract and pancreas. Pneumothorax is a rare complication of ERCP. We report two cases of pneumothorax following elective ERCP for ductal stone clearance. The first patient was a 45-year-old female, who developed respiratory distress, abdominal pain, and profoundly abdominal distention immediately after the procedure. Imaging studies revealed the presence of a right-side pneumothorax, pneumomediastinum, pneumoperitoneum, and pneumoretroperitoneum. The second patient was a 94-year-old female, who developed tension pneumothorax with clinical signs of shock during the procedure. Imaging studies revealed the presence of a right-side pneumothorax without free air in the mediastinum and retroperitoneal space. The imaging findings suggest that the occurrence of this rare complication in our patients was caused by entirely different pathophysiological mechanisms. Both patients were successfully treated with chest tube insertion, and no further intervention was required. Clinicians should be aware of this serious complication because delayed diagnosis may involve significant morbidity and mortality risks.

## 1. Introduction

The widespread use of ERCP has changed the management of many patients with biliary and pancreatic disease. ERCP is an interventional procedure which accompanies complications [1].

We report two rare cases of pneumothorax complicating ERCP, which were treated conservatively. Pathophysiological mechanisms underlying ERCP-related pneumothorax in these two cases were entirely different. In the first case, air enters the retroperitoneal space after interruption of the duodenal barrier due to a deep sphincterotomy. Subsequently, the air transfers from the retroperitoneal space to the mediastinum, where after a rupture of the parietal pleura passed to the pleural cavity. In the second case, the most likely pathophysiological mechanism was an alveolar rupture due to increased intrathoracic pressure maybe because of Valsalva manoeuvre during the procedure. 

## 2. Case Presentations

### 2.1. Case 1

**Figure 1:**
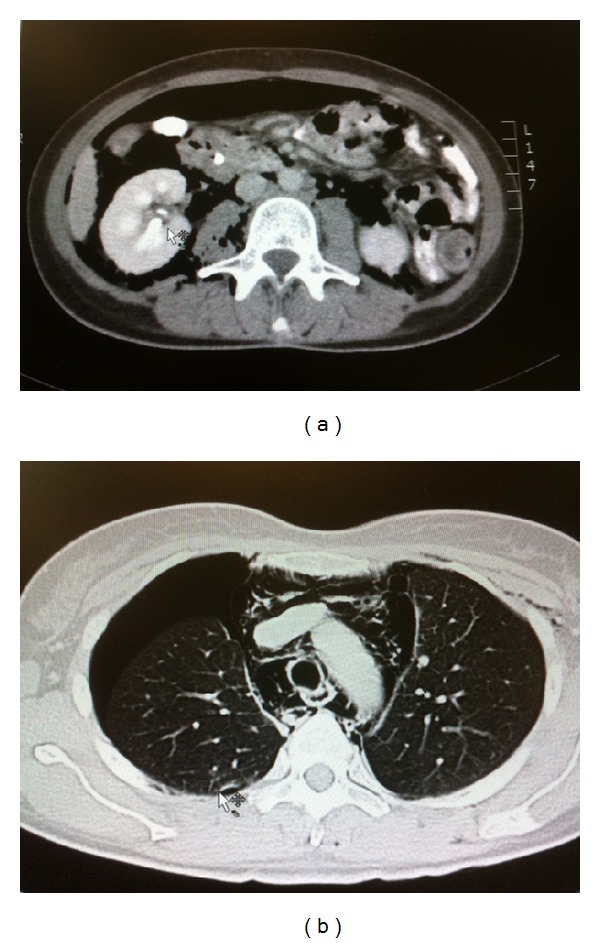
(a) Abdominal CTscan showing intra- and retroperitoneal free air. (b) Thoracic CT showing right-sided pneumothorax and presence of mediastinal air.

A 45-year-old female patient was admitted for ductal stone clearance. She underwent laparoscopic cholecystectomy 1 month ago. During the postoperative period, she developed obstructive jaundice. An abdominal ultrasound scan showed a distended common bile duct containing big calculi. During ERCP, a guide wire-assisted sphincterotomy was performed, and two big common bile duct stones were removed. Immediately after the procedure, patient developed abdominal distension, abdominal pain, dyspnoea, and chest pain. Physical examination revealed diminished breath sounds on the right side of the chest. The abdomen was distended although remained soft and without peritoneal signs. Chest X-ray revealed extensive right-side pneumothorax and pneumomediastinum. Abdominal X-ray revealed pneumoperitoneum and pneumoretroperitoneum. Oxygen saturation was 95%. The patient was resuscitated and taken directly for a chest and abdominal computed tomography scan. CT confirmed the presence of pneumoperitoneum, pneumoretroperitoneum, pneumomediastinum, and right-side pneumothorax (Figure 1). No extraluminal contrast medium was revealed. 

**Figure 2:**
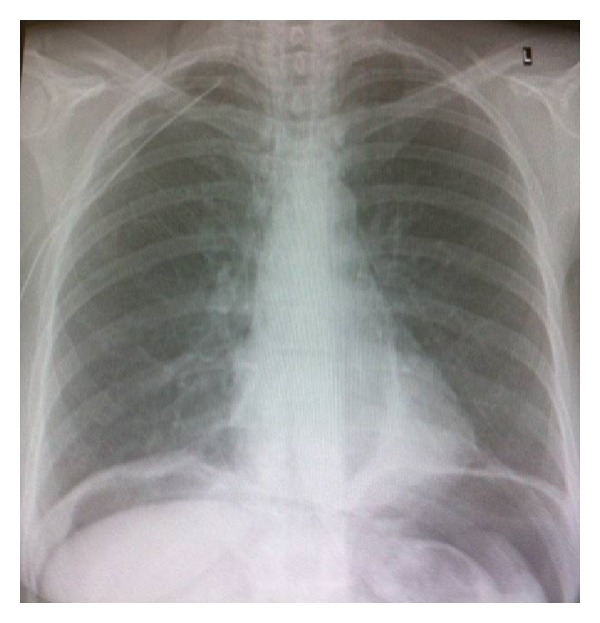
Chest X-ray after the insertion of a right-sided chest tube. Pneumothorax has completely resolved. Existence of Intraperitoneal free air.

The patient was managed conservatively with insertion of a right-sided chest tube, intravenous antibiotics, and fasting. By the fourth day of admission, the pneumothorax had totally resolved (Figure 2), and chest drain was removed. The patient was discharged the following day.

### 2.2. Case 2

**Figure 3:**
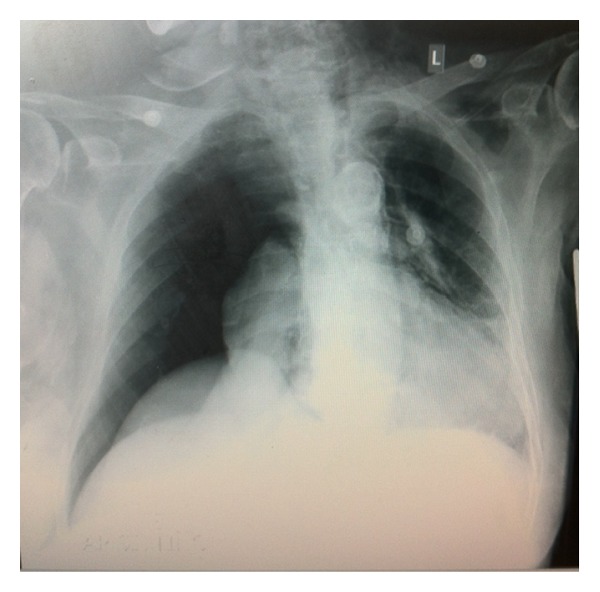
Chest X-ray showing right-sided pneumothorax. There is no intraperitoneal free air.

A 94-year-old female patient was admitted because of biliary colic and intermittent obstructive jaundice. She was scheduled for ERCP for removal of biliary stones. During the procedure, the patient was exceedingly anxious. Immediately after cannulation of the papilla of Vater, patient developed dyspnoea, chest pain, and clinical signs of shock. Her blood pressure was 80/40 mmHg and her heart rate 140. We stopped the procedure. Physical examination revealed diminished breath sounds on the right side of the chest. Chest X-ray revealed extensive right-side pneumothorax without pneumomediastinum (Figure 3). In contrast with the previously described case, there were no abdominal symptoms, and there were no intraperitoneal free air. After the insertion of a right-sided chest tube, the symptoms retreated. We did not proceed in further imaging because of the age of the patient. 

The patient was managed conservatively only with the chest tube. She was fed the same day. Three days later, we repeated the ERCP, and this time with the patient was intubated. A guide wire-assisted sphincterotomy was performed, and many small common bile duct stones were removed. The chest tube was removed after 7 days, and the patient was discharged the following day.

## 3. Discussion

ERCP is a routine procedure in the diagnosis and treatment of biliary and pancreatic diseases. ERCP is a technically demanding procedure with a considerable potential for serious complications. Common complications are pancreatitis, cholangitis, hemorrhage, and perforation [2]. The rate of complications reportedly ranges from 5 to 6.9%, with a mortality rate of 0.33% [3, 4]. ERCP-related perforation is the most serious complication, with a high mortality rate [5, 6].

Complications such as pneumothorax, pneumomediastinum, pneumoperitoneum, and pneumoretroperitoneum after ERCP are rare [7–10]. The main risk factors, for ERCP-related pneumothorax, are (precut) sphincterotomy and possibly the presence of juxtapapillary diverticula [11].

During ERCP, air can reach pleural cavity through three different ways. In most cases, pneumothorax coexists with pneumoretroperitoneum [10, 12]. This finding indicates that air enters the retroperitoneal space after interruption of the duodenal barrier, through a site of perforation or a site of low resistance [13–15]. Subsequently, air transfers from the retroperitoneal space to the peritoneum, subcutaneous tissue, mediastinum, and finally pleural space. Passage of air from the mediastinum to the pleural space demands a rupture of the parietal pleura. Sphincterotomy is the main cause of retroperitoneal perforation. The retroperitoneal perforation in turn causes the accumulation of air to the retroperitoneal space. This type of perforation varies from peritoneal perforation to the clinical signs. The absence of leakage of intestinal contents in peritoneal cavity results in the lack of peritoneal signs. 

Development of pneumothorax through this pathophysiological mechanism suits our first case where we confirmed the presence of free air to the retroperitoneal space, intraperitoneal, mediastinum, and pleural cavity. Additionally, the lack of peritoneal signs in this patient suggests that the perforation was towards retroperitoneal space.

Two alternatives, but less possibly pathways are through pores in the diaphragm [16] or alveolar rupture. We believe that the development of pneumothorax in the second patient was due to alveolar rupture. The patient during the procedure was extremely anxious with a continuous retching. We believe that the patient's response was the equivalent of intensive and continuous Valsalva manoeuvres. These manoeuvres increased the intrathoracic pressure and eventually drove to alveolar rupture and the development of tension pneumothorax. In agreement with the above hypothesis regarding the mechanism of development of tension pneumothorax to our second case are the absence of free air in retroperitoneal space, intraperitoneal cavity, and mediastinum.

As we mentioned previously, this patient developed clinical signs of shock during the ERCP. Although it turned out that the cause of these symptoms was tension pneumothorax, initially the differential diagnosis included the possibility of systemic air embolism. This rare complication has been described in the literature as a result of endoscopic interventions [17]. Air can pass from the alimentary track or the biliary system to the surrounding blood vessels through defects of the barriers of these hollow organs. The insufflation of air during endoscopic procedures, like ERCP, augments the possibility of the passage of air to the blood vessels. At the same time, many of the interventions during ERCP and mainly sphincterotomy affect the integrity of duodenal or biliary system mucosal barrier. This in turn further increases the likelihood of passage of air into the surrounding blood vessels. In most of the cases, the air remains in the portal vein causing portal venous air embolism which is absorbed spontaneously. However, serious complications have occurred when the air enters to the systemic circulation causing systemic air embolism with fatal outcome in some cases [18, 19].

The clinical presentation of pneumothorax depends on the amount of air that escaped from duodenal lumen. Most common signs and symptoms are abdominal distention, abdominal pain, chest pain, dyspnoea, and subcutaneous emphysema [20].

Both CT and X-rays can reveal free air in retroperitoneum, peritoneal cavity, mediastinum, and pleural cavity. Abdominal CT scan with contrast medium administration is fundamental regarding diagnosis of duodenal perforation. Surgical indications after duodenal perforation are acute peritoneal irritation signs with or without sepsis, documentation of large contrast extravasation, and presence of intra- or retroperitoneal fluid collections. 

Obviously, because of the rarity of this complication, there are no large series or controlled studies with respect to the optimal treatment of this entity. However, the increasing number of case reports indicates that a nonsurgical approach can be followed [11]. Conservative treatment consists of administration of intravenous antibiotics, fasting, and pleural drainage. When the initial conservative treatment strategy of retroperitoneal perforation is not successful, surgical closure of the leak is the treatment of choice [21]. In extremely rare cases where the development of pneumothorax is not due to air leakage from the duodenum, like our second patient, we believe that fasting is not necessary.

## 4. Conclusion

Pneumothorax is a rare but potentially lethal complication of ERCP. Although surgical intervention to address retroperitoneal perforation is not excluded, in most cases, the conservative treatment is sufficient to address this complication. The most crucial part of the management of these patients is the early detection of this rare complication.
